# Two distinctly located primary orbital melanomas following evisceration for congenital corneal staphyloma: a case report and brief literature review

**DOI:** 10.3389/fmed.2025.1662161

**Published:** 2026-01-05

**Authors:** Chaona Zhou, Pengsen Wu, Fangwei Ying, Jing Rao, Yanlin Wen, Guiqin Liu

**Affiliations:** 1 The Third People’s Hospital of Longgang, Clinical Institute of Shantou University Medical College, Shenzhen, China; 2 Shenzhen Eye Hospital, Shenzhen Eye Medical Center, Southern Medical University, Shenzhen, China; 3 National Cancer Center/National Clinical Research Center for Cancer/Cancer Hospital & Shenzhen Hospital, Chinese Academy of Medical Sciences and Peking Union Medical College, Shenzhen, China

**Keywords:** primary orbital melanoma, blue nevi, evisceration, congenital corneal staphyloma, prosthesis

## Abstract

**Purpose:**

To report an unusual case of two distinct primary orbital melanomas emerged 9 years following evisceration for congenital corneal staphyloma and implantation of a MEDPOR^®^ prosthesis.

**Case description:**

A 14-year-old female with congenital blindness and progressive right globe enlargement since birth, accompanied by multiple cutaneous blue nevi, initially underwent right eye evisceration with MEDPOR^®^ implant placement in 2012, revealing congenital corneal staphyloma and proliferative vitreoretinopathy on histopathology. Nine years later, she presented with chronic right eye tearing and intermittent bloody discharge. Examination revealed a prominent scalp blue nevus with satellite lesions, mild orbital implant protrusion, conjunctival congestion, and a subconjunctival black mass. MRI identified a 1.9 cm T1-slight hyperintense, T2-hypointense mass superior to the implant, with inferior rectus thickening. Surgical excision yielded two masses: a 41 mm inferonasal lesion (partially pigmented, unencapsulated) and a 20 mm superior–posterior black, spherical mass. Histopathology confirmed orbital malignant melanoma, with tumor cells positive for S-100 (3+), Melan-A (3+), and HMB-45 (2+). NGS revealed no pathogenic variants, and PET/CT showed no metastasis. She subsequently underwent postoperative radiotherapy and maintains good overall health until June 2025.

**Discussion:**

This article reports a case of primary orbital melanoma presenting as two tumors within a single orbit following evisceration, with a focus on elucidating the origin of tumor cells and their relationship with pigmented nevi and orbital implants.

**Conclusion:**

Blue nevi may represent an underrecognized risk factor for primary orbital melanoma, and enucleation may be preferable to evisceration for idiopathic corneal staphyloma to reduce melanoma risk.

## Introduction

Orbital melanoma is classified into three types based on its origin: primary (arising *de novo* within the orbital tissues), secondary (resulting from direct invasion by adjacent tumors, such as uveal or conjunctival melanoma), and metastatic (originating from hematogenous dissemination of distant primary melanomas) (1). Among these, primary orbital melanoma (POM) represents an exceedingly rare orbital malignancy, accounting for merely 1% of all orbital tumors (2). To date, fewer than 100 cases have been documented (3, 4). Regarding the origin of tumor cells of POM, about 39–90% are associated with blue nevus or congenital melanosis. It has also been reported that it originates from the orbital residual neural crest cells, ciliary nerves, scleral emissary veins, the leptomeninx of the optical nerve, or ectopic melanocyte nests in the orbit (3, 5–7).

Upon reviewing the literature, it was observed that the majority of POM cases exhibited unilateral single lesions, whereas unilateral multiple lesions were exceedingly rare. This case report aims to present a rare instance of POM manifesting as two tumors within a single orbit.

We present a case of congenital corneal staphyloma associated with a blue nevus, in which two distinct primary orbital melanomas emerged 9 years following evisceration and implantation of a MEDPOR^®^ prosthesis. To the best of our knowledge, this represents the first documented case of primary orbital melanoma following evisceration of a congenital corneal staphyloma, and it is also the sole reported instance of two melanomas occurring within a single orbit when first identified.

### Case description

**Figure 1:**
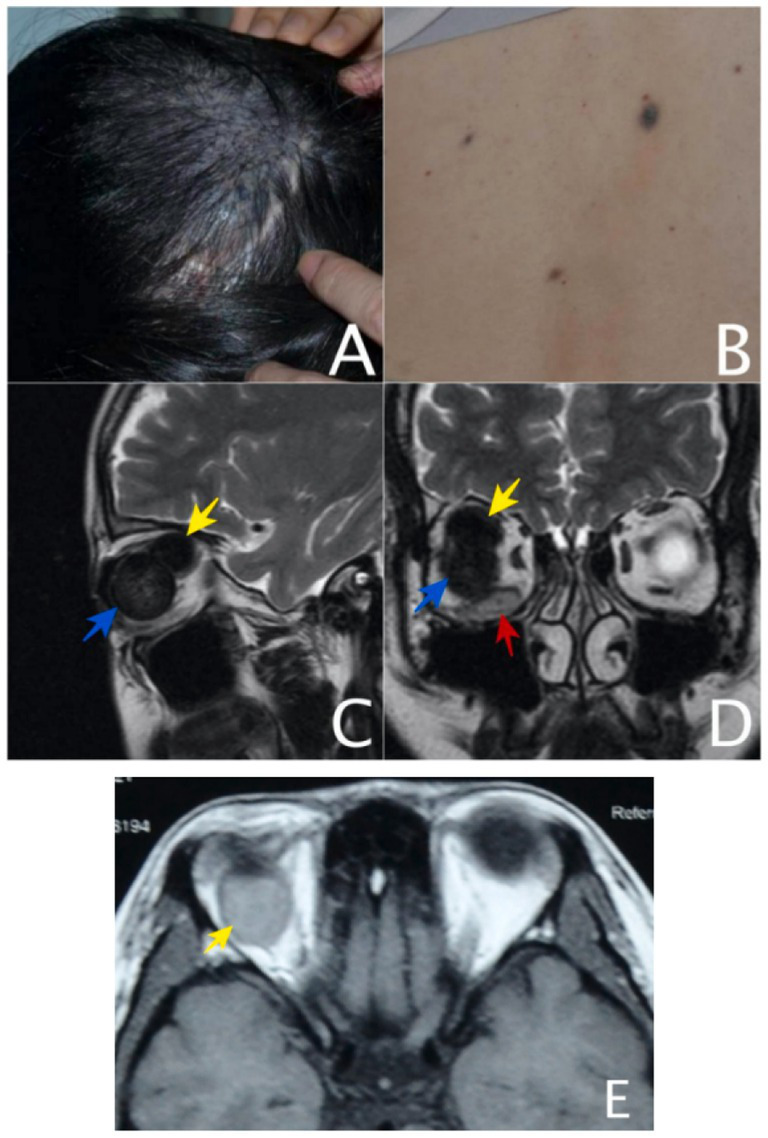
**(A)** A large blue nevus on the right upper scalp. **(B)** Small blue nevi on waist skin. **(C)** MR images on T2WI: Sagittal scan of the right orbit: showed an oval-shaped, homogeneous hypointense mass (the yellow arrow) relative to extraocular muscle above and behind the orbital implant (the blue arrow), the right inferior rectus muscle significantly thickened and slight hyperintense signal. **(D)** MR images on T2WI: coronal scan: showed an oval-shaped, homogeneous hypointense mass (the yellow arrow) relative to extraocular muscle above and behind the orbital implant (the blue arrow), the right inferior rectus muscle (the red arrow) significantly thickened and slight hyperintense signal. **(E)** MR images on T1WI: horizontal scan: showed an oval-shaped, homogeneous slight hyperintense mass (the yellow arrow) relative to extraocular muscle and behind the orbital implant (the blue arrow).

On July 2012, a 14-year-old female patient was referred to our institution with a history of congenital blindness and progressive, painless enlargement of the right globe noted since birth. Multiple cutaneous blue nevi were observed, including a prominent lesion (approximately 4.5 cm in diameter) on the right parietal scalp, with smaller satellite nevi distributed across the lumbosacral region and right lateral thigh. All lesions exhibited slow, progressive enlargement correlating with patient growth. Ophthalmic evaluation revealed no light perception (NLP) in the right eye, while the left eye demonstrated normal visual acuity (20/20). The right globe demonstrated significant pathological features, including corneoscleral enlargement with digital intraocular pressure measurement of T + 1, inferior corneal degeneration accompanied by calcific deposits, prominent scleral vascular engorgement, posterior synechiae formation at nasal and inferior quadrants. Ocular examination revealed a shallow anterior chamber (5CT) and a fixed pupil measuring 5 mm in diameter. The lens showed complete opacification. B-scan ultrasonography revealed extensive vitreous opacities with diffuse thickening of the globe wall. It was not possible to distinguish between the retina and the choroid. Following comprehensive evaluation, the patient underwent successful evisceration of the right eye under local anesthesia with implantation of a MEDPOR^®^ Surgical Implant Sphere (Porex Surgical Inc., USA). Histopathological examination confirmed the presence of corneoscleral staphyloma and proliferative vitreoretinopathy. Simultaneously, retinal degeneration and edema were observed, with localized calcification noted; the choroid was thickened and fibrotic, with focal calcific deposits present. However, the patient was readmitted on July, 2021, reporting chronic right eye tearing and intermittent bloody discharge for 9 years, with progressive worsening over the past 2 years. Physical examination: a dome-shaped, walnut-sized blue nevus was noted on the right scalp, along with over a dozen smaller blue nevi (ranging from pinhead- to rice-sized) scattered across the lower back and lateral aspect of the right thigh (Figures 1A,B). No palpable lymphadenopathy was noted. The right ocular implant is in position. The conjunctiva exhibits mild hyperemia, with two flat, pigmented lesions observed in the subconjunctival space inferonasally. The left eye was unremarkable. MRI revealed a 1.9 × 1.5 × 1.5 cm oval mass superior to the right orbital implant, displaying homogeneous slight hyperintensity on T1WI and hypointensity on T2WI relative to extraocular muscles, with concomitant thickening of the inferior rectus muscle (slightly hyperintense on T2WI). Based on the integration of clinical presentation and MRI findings, the case was stratified as localized high-risk disease. The patient subsequently underwent surgical intervention involving resection of the orbital mass and explanation of the orbital implant. Intraoperatively, two masses were excised: a 41 × 23 × 8 mm mass beneath the implant and a 20 × 18 × 17 mm mass above it during implant removal (Figures 1C–E). The lower mass was partially black and partially flesh-colored, elongated in shape, without a capsule, with clear boundaries and a firm texture. The cut surface of the mass was partially gray-black and partially flesh-colored, with a texture resembling fish meat.

**Figure 2:**
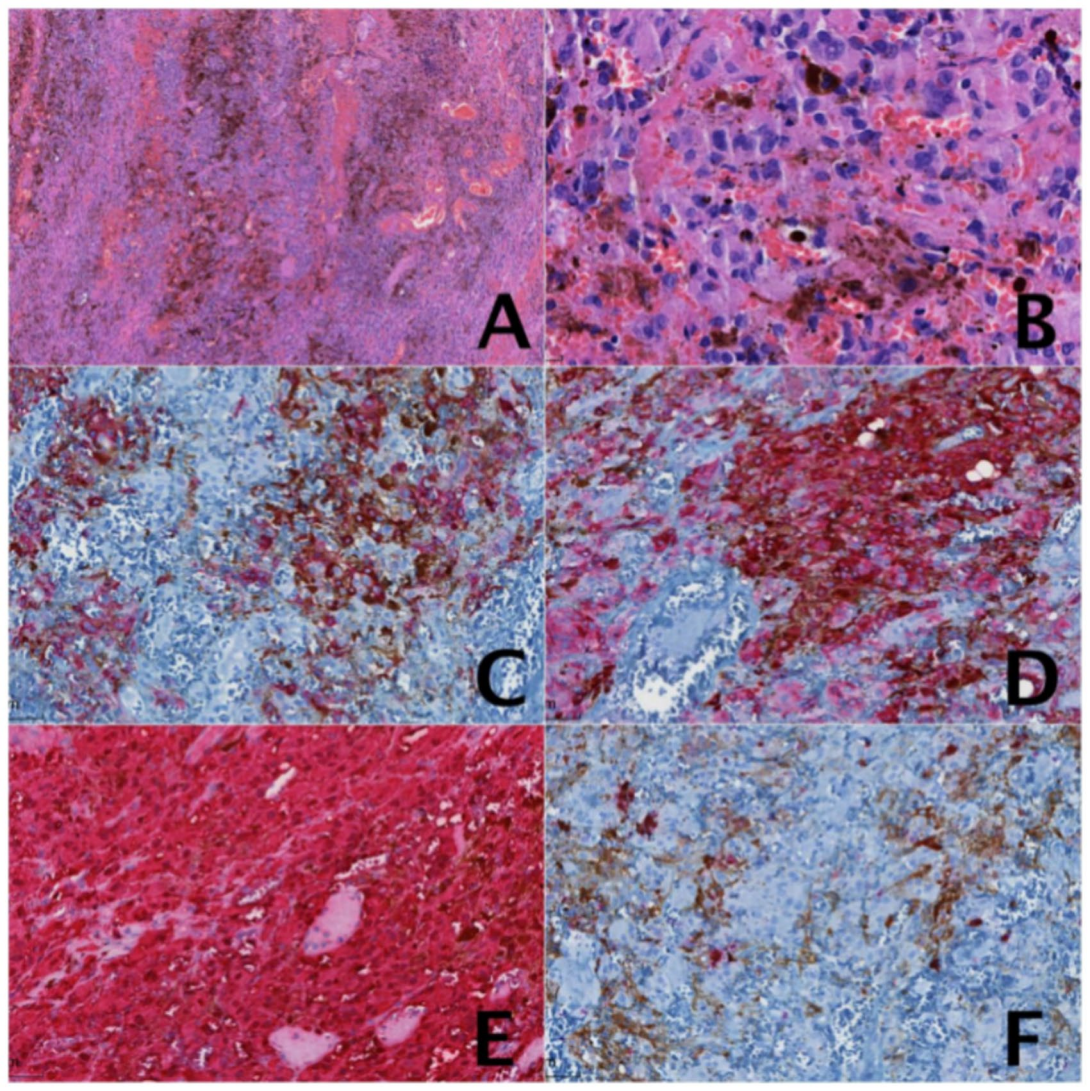
**(A)** The tumor cells were distributed in sheets within the tissue, with oval or elongated nuclei and pigment-rich cytoplasm. The interstitium was rich in blood vessels. HE staining, 4×. **(B)** Pigment-rich tumor cells with oval, oblong nuclei. HE Staining, 40×. **(C)** Immunohistochemical examination: HMB-45 (2+) 20×. **(D)** Immunohistochemical examination: Melan-A (3+) 20×. **(E)** Immunohistochemical examination: S-100 (3+) 20×. **(F)** Immunohistochemical examination: Ki-67 (about 5%+) 20×.

The lower mass appeared partially black and partially flesh-colored, exhibiting an elongated shape without a capsule. It had well-defined margins and a firm consistency. On sectioning, the mass displayed a variegated cut surface-partly gray-black and partly flesh-colored-with a fish meat-like texture. The upper mass was black, hard, and roughly spherical in shape. The cut surface was entirely black, with a texture resembling fish meat. Pathological examination: right orbital malignant melanoma. Immunohistochemical examination: S-100 (3+), Melan-A (3+), HMB-45 (2+), GFAP (−), Syno (−), Ki-67 (about 5%+) (Figure 2). Pathological diagnosis: right orbital malignant melanoma. Comprehensive next-generation sequencing (NGS) testing was performed, with results showing an absence of pathogenic variants associated with clinical phenotype. Postoperative PET/CT revealed (1) post-surgical changes following resection of the right orbital mass; (2) thickening of the subcutaneous soft tissue in the right frontoparietotemporal region, accompanied by hypermetabolism, suggestive of a fibroma; (3) possible teratoma. Consistent with the multidisciplinary tumor board’s assessment involving specialists from radiology, pathology, medical oncology, and radiation oncology, the patient’s orbital melanoma remains classified as localized high-risk disease. Six weeks after operation, at the advice of the oncology department, she underwent adjuvant radiation therapy with a total dose of 67.5 Gy in 27 fractions of 2.5 Gy each.

**Figure 3:**
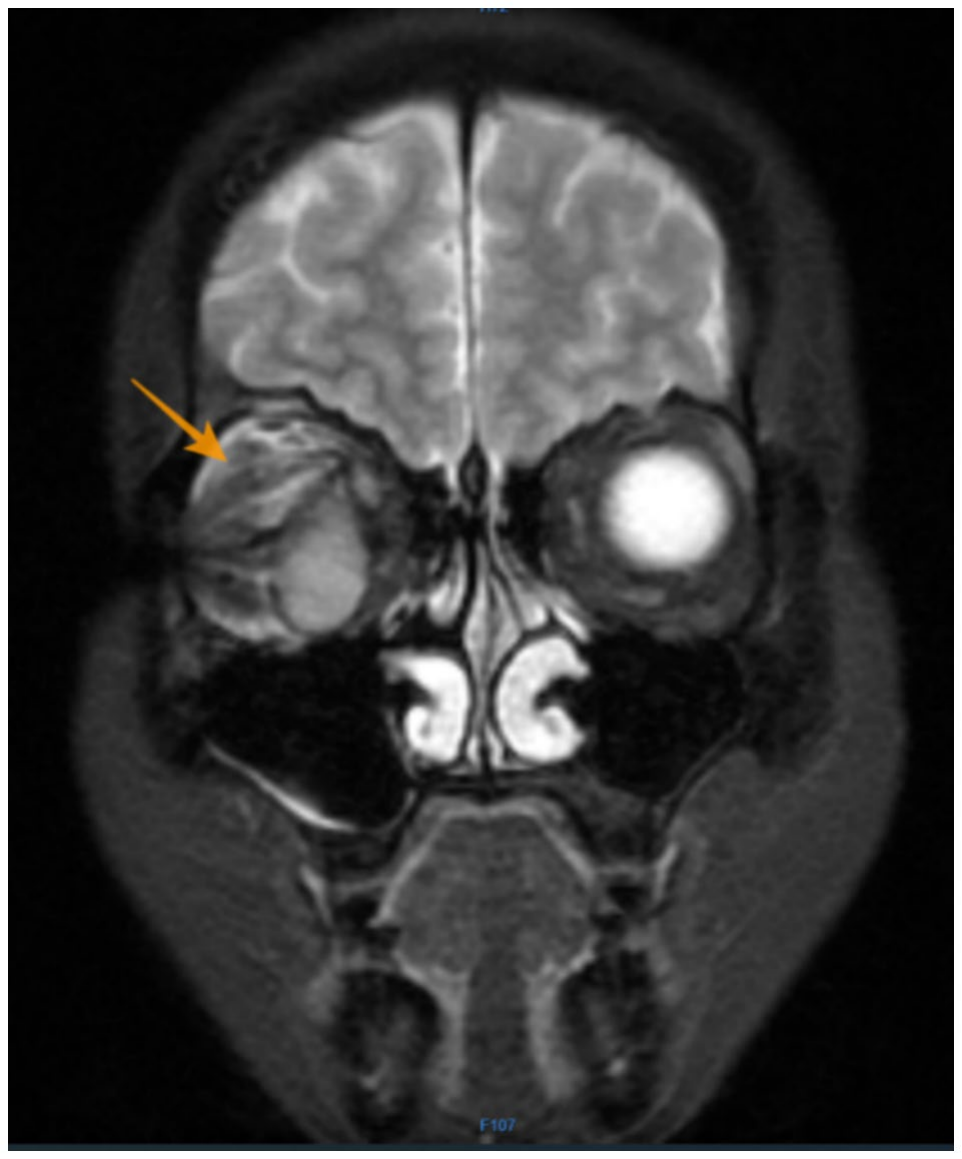
Orbital MRI reexamination on July, 2024: structural disorganization of the soft tissues in the periorbital space, with patchy mixed high signal intensity on T2WI and irregular enhancement. The lesion demonstrates ill-defined margins with the superior and lateral rectus muscles, measuring approximately 1.5 × 0.9 cm (the orange arrow).

Follow-up: The patient maintained good overall health for 3 years after her second surgery but subsequently developed right upper and lower eyelid atrophy with conjunctival sac closure, precluding ocular prosthesis use. Orbital MRI reexamination on July 25, 2024, revealed postoperative changes following resection of malignant melanoma in the right orbit: abnormal morphology of the right ocular prosthesis. Subcutaneous fascial swelling adjacent to the right orbit: structural disorganization of the soft tissues in the periorbital space, with patchy mixed high signal intensity on T2WI and irregular enhancement. The lesion demonstrates ill-defined margins with the superior and lateral rectus muscles, measuring approximately 1.5 × 0.9 cm (Figure 3). In August 2024, she underwent conjunctival sac reconstruction using lip mucosa transplantation, posterior eyelid reconstruction with hard palate mucosa transplantation, and eyelid margin adhesion surgery, with planned adhesion release 1 year later for prosthetic fitting. Regarding the blue nevus on the patient’s scalp and back, there have been no significant changes in size or color, and no new symptoms have emerged.

## Discussion

Primary orbital melanoma (POM) is a rare orbital malignant tumor (2). Its incidence is only about 1% in orbital tumors and there are only about 100 cases reported at present (Appendix 1). In this case, the concurrent presentation of two malignant melanomas with distinct gross appearances in the same orbit is exceedingly rare and may imply differing origins.

The most common clinical manifestations of POM are varying degrees of progressive painless exophthalmos, which may be accompanied by vision changes, eyelid swelling, diplopia, incomplete eyelid closure, limited eyeball displacement and eye movement depending on the size and location of the tumor (3, 8). MRI serves as an effective diagnostic modality for melanoma due to melanin’s distinctive signal characteristics: hyperintense on T1-weighted sequences and hypointense on T2-weighted sequences (9). However, in amelanotic melanoma, the characteristic imaging presentation may be absent owing to the loss of melanin-derived paramagnetic effects (10). PET-CT has unique advantages in showing whether there are primary diseases or metastases in other locations. The tumor is usually a black mass with clear or unclear boundaries, unencapsulated or incomplete pseudocapsule, and may be accompanied by surrounding bone erosion (11). In this instance, the tumor exhibits a biphasic coloration, with portions appearing flesh-colored and others black, a presentation that is considered uncommon. Tumor cells of different sizes and obvious atypia can be seen diffusely distributed in sheets or nests, and appear spindle-shaped or epithelioid. In addition, the cytoplasm contains varying amounts of melanin (12). Some studies believe that in adult patients with melanosis, the prognosis is poor when the tumor cells are epithelial and mitotic figures are common (4). By immunohistochemistry, the lesional cells demonstrated difuse positive staining with melanocytic markers including SOX10, Melan-A, S100, and HMB-45 (13). There is good correlation between MIB-1 reactivity in primary thick melanomas and metastatic potential and there is a association between loss of HLA class I and II antigens with a more aggressive nature of the tumor (14, 15). The primary treatment for primary orbital melanoma (POM) remains surgical intervention, with the two main approaches being local tumor excision and orbital exenteration. In addition to surgical management, adjuvant therapies such as radiotherapy and chemotherapy may also be incorporated into the treatment regimen (16). POM is generally characterized as a highly malignant disease with an unfavorable prognosis. Nonetheless, there are documented cases of patients exhibiting prolonged survival, including those with hepatic metastases (4, 13).

Although pigment cells are generally considered to be predominantly localized in the anterior uvea and retina, with melanocytes typically absent in orbital soft tissues, Werf et al. (17) previously identified pigmented cells (PCs) in extraocular tissues—such as the sclera, Tenon’s capsule, and periscleral tissues between the sclera and bony orbit—in both humans and crab-eating monkeys. Consequently, melanocytes in the orbit may originate from ectopic melanocytic nests within the orbital region.

Melanocytes derive from neural crest cells, which undergo differentiation and migration to various bodily regions. Normally, neural crest cells delaminate from the neuroectoderm, transitioning from neuroepithelial to mesenchymal cells. While most contribute to craniofacial structures such as bones, muscles, and dermis, a subset migrates through the dermis and differentiates into melanocytes (18). Therefore, potential sources of POM tumor cells may include residual orbital neural crest cells, ciliary nerves, scleral emissary veins, the leptomeninges of the optic nerve, or ectopic melanocytic nests in the orbit.

This case warrants special consideration given the patient’s history of evisceration, which presents two clinically significant implications. First, residual uveal tissue within the orbit—demonstrated as a potential source of melanocytes in Murthy’s case report—may persist following this procedure. Second, the surgical intervention itself could theoretically induce malignant transformation of melanocytes through chronic inflammation or microenvironmental alterations.

Additionally, while no direct evidence links orbital implants to POM development, Ferreira et al. (9) reported a POM case following enucleation, and Quhill et al. (19) described a 16-year-old anophthalmic patient developing a conjunctival blue nevus 5 years post-enucleation. They hypothesized that hormonal influences or implant-related stimulation might contribute to nevus formation. Thus, the potential role of the orbital implant in POM pathogenesis in this case warrants consideration.

Blue nevus and cellular blue nevus are benign, intradermal melanocytic tumors that arise from unregressed neural crest cells. The neural crest cells embryonically migrated along developing nerves. The arrest of neural crest melanocyte precursors within the dermis can cause blue nevus (20, 21). Congenital melanosis, which includes the periorbital pigment disorders ocular melanocytosis and the nevus of Ota, also occurs from arrested migration of melanocytes (21). Histologically, blue nevi consist of dendritic melanocytes localized to the dermis, sparing the epidermis. Their characteristic blue appearance stems from the Tyndall effect, as deeper melanin pigment scatters shorter wavelengths of light. These lesions most commonly occur on the dorsal hands, feet, scalp, and buttocks. Oculodermal melanocytosis (nevus of Ota) shares a similar pathogenesis but exhibits less densely aggregated melanocytes compared to classic blue nevi (16).

Approximately 39 to 90% of POM have been reported to be associated with blue nevi or congenital melanosis (3). Orbital melanoma associated with melanocyte proliferation in blue nevi has been documented as early as 1954 (22). Previous studies have reported that the presence of blue nevus supports the classification of orbital melanoma as a primary rather than metastatic lesion, given that blue nevus cells are considered precursors to orbital melanoma (20). In Roelofs’s case report (23), the affected ocular region in this patient demonstrated histopathological features consistent with melanocytosis, including localized proliferation of melanocytes, cellular atypia, and scleral stromal dilation—morphological characteristics resembling those of a blue nevus. Based on these findings, the authors suggest that blue nevi may represent an intermediate pathological stage in the potential progression from ocular melanocytosis to melanoma. This case of primary orbital melanoma (POM) is unique for its presentation of “two distinct tumors in one orbit” characterized by separate locations and different morphological features. Literature review reveals that multifocal POM manifestations are exceptionally rare. In 2021, Claxton et al. (24) reported a case featuring three distinct tumors in a unilateral orbit—two diagnosed as melanomas and one as melanocytoma—with these tumors demonstrating varying degrees of cellular atypia and genetic alterations.

In the present case, the orbital melanoma is considered primary and potentially associated with blue nevus syndrome. The tumor may originate from conjunctival melanocytes, ectopic melanocytic nests within the orbit and extraocular muscles, or residual melanocytes following prior surgical intervention. The patient presented with two morphologically distinct tumors. The first tumor, located above and posterior to the orbital implants, appeared as a round, black mass composed entirely of neoplastic tissue. The second tumor was strip-shaped and situated below the orbital implant. This lower mass exhibited a partly flesh-colored and partly black appearance, representing an enlarged, malignant inferior rectus muscle.

The pathological examination revealed that the inferior tumor exhibited an admixture of melanocytic cells and muscular tissue, while the tumor cells in both lesions demonstrated consistent morphological features and immunophenotypic profiles. This discrepancy suggests distinct origins of the two tumors: the superior tumor likely arose from the intraorbital melanocytic nests, whereas the inferior tumor may have originated from melanocytic nests within the extraocular muscles. In the latter, some muscular tissue had undergone complete malignant transformation.

Chiou et al. (25) previously reported a case of POM potentially originating from scleral melanocytes. However, in the present case, given that the tumor location showed no adhesion to the scleral wall, the likelihood of its derivation from scleral melanocytes appears to be low.

Another distinctive aspect of this case is that the patient underwent eyeball evisceration followed by orbital implantation at the age of 14 due to congenital corneal staphyloma. To date, no studies have explored the association between congenital corneal staphyloma and POM. Since histopathological examination of the enucleated ocular tissue revealed no tumor cells, we postulate that the congenital corneal staphyloma in this patient was attributable to anterior segment dysplasia rather than childhood intraocular melanoma.

This case emphasizes that in congenital corneal staphyloma of unknown etiology, evisceration should be performed with caution to avoid the possible residual of melanocytes or even tumor cells.

Limitations: (1) No pathological examination was performed on the blue nevus of the scalp and lumbar region. When the patient was previously seen in the plastic surgery department, the physician had planned to excise the scalp pigmented lesion, but this was ultimately abandoned due to the excessively large size of the lesion. After the diagnosis of primary orbital melanoma, we also suggested a biopsy of the blue nevus, but this was refused by the patient and her family members. (2) No orbital imaging was performed prior to the evisceration. Although evisceration was not performed for oncological reasons, for this specific case, this remains a regrettable oversight.

In conclusion, we report a rare case of primary orbital melanoma presenting as two distinct masses in a adolescent female with unilateral congenital corneal staphyloma and blue nevus syndrome (scalp and lower extremities), occurring 9 years post-enucleation. The diagnosis was established based on: (1) MRI for definitive tumor localization and extent; (2) pathological biopsy and immunohistochemistry confirming melanoma; (3) Ocular content histopathology combined with PET-CT verification of tumor primarity. Treatment involved complete tumor excision with prosthesis removal and adjuvant radiotherapy. No recurrence was observed during 3-year follow-up. We propose these melanomas may originate from orbital muscle melanocytes or residual neural crest cells, potentially associated with her blue nevi. This case suggests: (1) blue nevi may represent an underrecognized risk factor for primary orbital melanoma, and (2) enucleation may be preferable to evisceration for idiopathic corneal staphyloma to reduce melanoma risk.

## Data Availability

The original contributions presented in the study are included in the article/Supplementary material, further inquiries can be directed to the corresponding author.
