# Supplementary material for: Two distinctly located primary orbital melanomas following evisceration for congenital corneal staphyloma: a case report and brief literature review
Source: Front Med (Lausanne). 2026 Jan 5;12:1662161. doi: 10.3389/fmed.2025.1662161 (PMC12813066; doi:10.3389/fmed.2025.1662161)
Supplement: Supplementary file 1 [file Table_1.docx]

**Appendix 1 Review of cases of primary orbital melanoma**

| Case | Author(s) (yr) | Number  of cases | Gender | Age of onset | Laterality | Clinical Presentation | Associated Lesion | origin of tumor | Treatment | Follow up | | | References |
| --- | --- | --- | --- | --- | --- | --- | --- | --- | --- | --- | --- | --- | --- |
|  |  |  |  |  |  |  |  |  |  | Time | Consequence | Alive |  |
| 1 | Acar^[17]^  （2021） | 1 | F | 28 | L | Blue–gray coloration of left periorbital skin and lower part of conjunctiva, proptosis | nevus of Ota | N/S | N/S | 20 m | metastasis | Y | Acar A, Palamar M, Bayraktaroglu S, Akalın T, Karaarslan I. Primary orbital  melanoma associated with nevus of Ota. Int J Dermatol. 2022 Apr;61(4):e141-e143. |
| 2 | Adetunji^[4]^（2021） | 1 | M | 38 | L | Proptosis&diplopia | no evidence of oculodermal melanosis, conjunctival melanoma, or uveal melanoma | N/S | proton beam radiation therapy+orbital exenteration | 5 m | no residual disease | Y | Adetunji MO, McGeehan B, Lee V, Maguire MG, Briceño CA. Primary orbital  melanoma: A report of a case and comprehensive review of the literature. Orbit. 2021  Dec;40(6):461-469 |
| 3 | Alsuhaibani^[16]^（2016） | 1 | M | 60 | L | swelling above the left eyeball and whitening of the left upper eyelid lashes | acquired poliosis of the eyelashe | N/S | exenteration of the left orbit | 6 m | no signs of local recurrence or distant metastasis | Y | Alsuhaibani AH, Alhumayed M. Primary orbital melanoma with poliosis and a  palpable mass. Arch Ophthalmol. 2011 Oct;129(10):1382-3. |
| 4 | Bains^~~[~~45]^（2016） | 1 | F | 28 | L | proptosis& decreased vision | calcification around the optic nerve | N/S | Lid sparing exenteration | About 34 m | Iecurrence&ntracranial metastasis | N | Bains S, Kim U, Shanti R. Orbital melanoma with calcification: A diagnostic  dilemma. Indian J Ophthalmol. 2016 Dec;64(12):932-934. |
| 5 | Buntinx-Krieg^[28]^（2016） | 1 | F | 45 | R | Diplopia, blurry vision,photophobia and pain | nevus of Ota | N/S | N/S | N/S | N/S | N/S | Buntinx-Krieg T, Ouyang J, Cartwright M. An Orbital Malignant Melanoma Arising in  Cellular Blue Nevus in a Patient with Nevus of Ota. Cureus. 2016 Jul 18;8(7):e698. |
| 6 | Chiou^[49]^（2024） | 1 | M | 16 | R | asymptomatic orbital mass | congenital ocular melanocytosis | intrascleral melanocytes | orbitotomy | 6 m | no recurrence or metastasis | Y | Chiou CA, Lin LY, Stagner AM, Lee NG. Melanoma Arising Beneath the Lateral  Rectus Muscle in a Teenager With Ocular Melanocytosis: Possible Origin From  Intrascleral Melanocytes. Ophthalmic Plast Reconstr Surg. 2024 Jul-Aug  01;40(4):e109-e111. |
| 7 | Claxton^[8]^（2021） | 1 | F | 33 | L | N/S | Oculodermal Melanocytosis | melanocytoma | orbitotomy with excisional biopsy | N/S | N/S | N/S | Claxton MR, Dalvin LA, Guo R, Tooley AA. Genetic Alterations in Melanocytoma  Associated with Oculodermal Melanocytosis: Molecular Characteristics. Ophthalmic  Plast Reconstr Surg. 2022 May-Jun 01;38(3):e77-e80. |
| 8 | Coppeto^[41]^（1978） | 1 | F | 49 | N/S | Painless visual loss& proptosis | CBN | CBN | Orbital exenteration | 2 y | no recurrence or metastasis | Y | Coppeto JR, Jaffe R, Gillies CG. Primary orbital melanoma. Arch Ophthalmol. 1978  Dec;96(12):2255-8. |
| 9 | Delaney^[12]^（2004） | 1 | M | 40 | L | painless proptosis | no clinical evidence of ocular melanosis or BN syndorme | N/S | a lateral orbitotomy with excision biopsy,2months after,exenteration with preseveratio  of eyelids,postoperative radiotherapy and adjunctive interferon treatment | 5 m | Metastasis in the anterior chest wall and the liver | N | Delaney YM, Hague S, McDonald B. Aggressive primary orbital melanoma in a  young white man with no predisposing ocular features. Arch Ophthalmol. 2004  Jan;122(1):118-21. |
| 10 | Dickens^[48]^（2021） | 1 | M | 27 | L | mild pain and swelling | BN/melanocytos | blue naevus-like diffuse melanocytosis | orbital exenteration | 26 m | no recurrence or metastasis | Y | Dickens TA, Franchina M, Gajdatsy A, Mesbah Ardakani N. Primary orbital  melanoma arising in an atypical diffuse (plaque-like) blue naevus/melanocytosis: a case  report and review of literature. BMC Ophthalmol. 2021 Dec 9;21(1):425. |
| 11-12 | Dorsey^[50]^（1954） | 2 | F | 24 | L | N/S | Naevus of Ota | N/S | N/S | 36 m | metastases | N | DORSEY CS, MONTGOMERY H. Blue nevus and its distinction from Mongolian spot  and the nevus of Ota. J Invest Dermatol. 1954 Mar;22(3):225-36. |
|  |  |  | M | 16 | R | Subcutaneous mass | Naevus of Ota and BN | N/S | Excisional biopsy | N/S | N/S | N/S |  |
| 13-14 | Dutton^[42]^（1984） | 2 | M | 15 | N/S | Orbital mass with increased overlying hyperpigmentation | Oculodermal melanocytosis | N/S | Anteromedial orbitotomy with trans frontal orbitotomy for tumour recurrence Radical exenteration | 18 m | recurrence | Y | Dutton JJ, Anderson RL, Schelper RL, Purcell JJ, Tse DT. Orbital malignant  melanoma and oculodermal melanocytosis: report of two cases and rev |
|  |  |  | M | 67 | N/S | Proptosis | Oculodermal melanocytosis | N/S | Radical exenteration | 16 m | no recurrence or metastasis | Y |  |
| 15 | Elibol^[14]^（1995） | 1 | M | 79 | L | a 9 year history of progressive tumour of the left eye | No associated orbital BN or melanosis | N/S | N/S | 11 m | no Metastasis | Y | Elibol O, Yüksel N, Egilmez HR, Arici S, Mizrak B. A case of primary orbital  melanoma treated by local excision. Br J Ophthalmol. 1995 Dec;79(12):1146-8. |
| 16-18 | El-Sawy^[47]^（2014） | 3 | F | 9 | R | proptosis&swelling of the right lower eyelid | CBN | N/S | orbital exenteration+Postoperative orbital radiotherapy | 36 m | no recurrence or metastasis | Y | El-Sawy T, Bakhoum MF, Tetzlaff M, Nasser QJ, Prieto VG, Ivan D, Sniegowski MC, Yin VT, Pan C, Durairaj V, Esmaeli B. Primary orbital melanoma in association with  cellular blue nevus. Digit J Ophthalmol. 2014 Jul 14;20(3):35-40. |
|  |  |  | F | 54 | L | Proptosis | CBN | N/S | orbital exenteration+Postoperative orbital radiotherapy | 24 m | no recurrence or metastasis | Y |  |
|  |  |  | F | 44 | R | visual disturbances | CBN | N/S | orbital exenteration+Postoperative orbital radiotherapy | 24 m | no recurrence or metastasis | Y |  |
| 19 | Fernández-Nogueras^[58]^  （2025） | 1 | F | 48 | R | Proptosis&periorbital headache | nevus of Ota | N/S | excision surgery+Postoperative radiotherapy and immunotherapy when new tumor growth | 12 m | good tolerance and response to treatment | Y | Segura Fernández-Nogueras MV, Tirado Pascual M, Piñas Hormeño H, Alba Linero  C. Primary orbital melanoma in a patient with nevus of Ota. Arch Soc Esp Oftalmol (Engl  Ed). 2025 Apr 15:S2173-5794(25)00071-4. |
| 20 | Ferreira^[18]^（2019） | 1 | M | 59 | R | a mass growing within the right orbit | N/S | N/S | Biopsy only | N/S | N/S | N/S | Ferreira AF, da Silveira Filho LG, Dias EL. Primary orbital melanoma in an  anophthalmic socket. Radiol Bras. 2019 Sep-Oct;52(5):347-348. |
| 21-24 | Figueira^[39]^（2017） | 4 | M | 27 | L | N/S | bulbar scleral pigmentation | probably arising from a cellular blue cellular naevus | extended exenteration | N/S | N/S | N/S | Figueira E, Rajak S, McKelvie P, Kalantzis G, Ismail A, Gonzales M, James C, McNab A, Selva D. Primary orbital melanoma: a case series and literature review. Orbit. 2018 Oct;37(5):352-357. doi: 10.1080/01676830.2017.1423354. Epub 2018 Feb 1. Erratum in: Orbit. 2018 Oct;37(5):i. |
|  |  |  | F | 70 | R | Proptosis& Diplopia | Small foci of melanocytic cells in scattered sections of the orbit | N/S | Exenteration | N/S | N/S | N/S |  |
|  |  |  | M | 50 | L | proptosis | CBN | N/S | extended (lateral zygomatic bone) exenteration | N/S | N/S | N/S |  |
|  |  |  | F | 26 | L | diplopia, reduced left eye vision, morning headaches, subconjunctival haemorrhage, and periocular swelling | N/S | N/S | exenteration with an irradiated socket allograft and adjuvant radiotherapy | 2 y | no recurrence or metastasis | Y |  |
| 25 | Friedrich^[9]^  (2007) | 1 | M | 22 | R | Proptosis& Diplopia | No associated orbital BN | N/S | via a latero-cranial orbitotomy | 14 m | N/S | Y | Friedrich RE, Grzyska U, Schäfer H, Li L. Navigation-assisted resection of a primary  extraocular melanoma of the orbit. Anticancer Res. 2007 Jul-Aug;27(4A):1799-803. |
| 26 | Gerami^[26]^（2011） | 1 | F | 52 | L | Rapidly progressive loss of vision | nevus of Ota | N/S | N/S | 11 m | no recurrence or metastasis | Y | Gerami P, Pouryazdanparast P, Vemula S, Bastian BC. Molecular analysis of a case  of nevus of ota showing progressive evolution to melanoma with intermediate stages  resembling cellular blue nevus. Am J Dermatopathol. 2010 May;32(3):301-305. |
| 27 | Granter^[53]^（2001） | 1 | M | 36 | N/S | N/S | BN | N/S | N/S | 36 m | N/S | Y | Granter SR, McKee PH, Calonje E, Mihm MC Jr, Busam K. Melanoma associated  with blue nevus and melanoma mimicking cellular blue nevus: a clinicopathologic study  of 10 cases on the spectrum of so-called 'malignant blue nevus'. Am J Surg Pathol. 2001  Mar;25(3):316-23 |
| 28 | Hagler^[51]^（1965） | 1 | M | 57 | L | Proptosis、diplopia&loss of vision | Naevus of Ota, BN, CBN | N/S | Exenteration | 10 m | N/S | Y | Hagler WS, Brown CC. Malignant melanoma of the orbit arising in a nevus of Ota. Trans Am Acad Ophthalmol Otolaryngol. 1966 Sep-Oct;70(5):817-22. |
| 29 | Haskins^[36]^（2017） | 1 | F | 53 | L | vision loss and proptosis | no evidence of nevus of Ota or diffuse uveal melanoma | N/S | orbital exenteration with temporalis flap reconstruction&Radiotherapy | 3.5 y | no evidence of diseas | Y | Haskins CP, Nurkic S, Fredenburg KM, Dziegielewski PT, Mendenhall WM. Primary  orbital melanoma treated with orbital exenteration and postoperative radiotherapy: A  case report and review of the literature. Head Neck. 2018 Mar;40(3):E17-E20. |
| 30 | Huii^[20]^  （2021） | 1 | M | 68 | R | proptosis&impaired visual acuity | N/S | N/S | orbitotomy | N/S | N/S | N/S | Hui VWK, Lau TC, Ng LPW, Yuen HKL, Cheuk W. Primary orbital melanoma. Hong  Kong Med J. 2021 Jun;27(3):223.e1-223.e2. |
| 31 | Hussain^[55]^（2017） | 1 | M | 59 | L | Blurred vision, eyelid swelling and blind spot in visual feld | CBN | N/S | Orbitotomy | N/S | Possible metastases | Y | Hussain A, Sidiropoulos M, Das S, Munoz DG, Nijhawan N. Orbital cellular blue  nevus complicated by malignant melanoma. Can J Ophthalmol. 2017  Jun;52(3):e111-e113 |
| 32 | Ijiri^[19]^  （2000） | 1 | F | 5 | L | proptosis&left subconjunctival hemorrhages | a blue subconjunctival mass located at the lower inner side of the left orbit | N/S | local tumor excision&postoperative chemotherapy | 7 m | no recurrence or metastasis | Y | jiri R, Tanaka Y, Kato K, Sekido K, Sato H, Ito D. Primary orbital melanoma in a child. Med Pediatr Oncol. 2000 Aug;35(2):142-3. |
| 33 | Jakobiec^[21]^  （1974） | 1 | F | 42 | L | Proptosis& Diplopia | left upper eyelid had been dark and pigmented | presumably CBN | exenteration of the left orbit、partial frontal  sinusectomy | N/S | N/S | N/S | Jakobiec FA, Ellsworth R, Tannenbaum M. Primary orbital melanoma. Am J  Ophthalmol. 1974 Jul;78(1):24-39. |
| 34 | Jay^[24]^  （1965） | 1 | F | 64 | R | proptosis | nevus of Ota | N/S | N/S | 10 m | Metastasis | N | Jay B. Malignant melanoma of the orbit in a case of oculodermal melanosis. (Naevus  of Ota). Br J Ophthalmol. 1965 Jul;49(7):359-63. |
| 36 | Ke^[35]^  （2014） | 1 | F | 8 | R | exophthalmos，decreased vision, epiphora, and pain | N/S | Giant Divided Nevus | Exenteration& Radiotherapy and chemotherapy | 10 m | recurrence and metastasis | N | ]Ke Y, Ren X, Zhu L, Hao R, Song W, Liu X, He Y. Primary orbital melanoma  combined with giant divided nevus of the eyelid. J Craniofac Surg. 2014 Jan;25(1):e4-7. |
| 37 | Konstantinov^[29]^（2018） | 1 | F | 29 | L | Severe headache, blurred vision，vomiting | nevus of Ota | N/S | N/S | N/S | Metastasis | N/S | Konstantinov NK, Berry TM, Elwood HR, Zlotoff BJ. Nevus of Ota associated with a  primary uveal melanoma and intracranial melanoma metastasis. Cutis. 2018  Sep;102(3):E2-E4. |
| 38 | Korányi^[23]^（2000） | 1 | M | 29 | L | Proptosis | oculodermal melanocytosis | congenital orbital melanosis | orbitotomy&interferon alpha-2b | 3 y | no recurrence or metastasis | Y | Korányi K, Slowik F, Hajda M, Bánfalvi T. Primary orbital melanoma associated with  oculodermal melanocytosis. Orbit. 2000 Mar;19(1):21-30. |
| 39-41 | Krishnakumar^[22]^（2003） | 3 | F | 45 | N/S | jaundice, with elevated liver enzymes and hepatic metastasis | N/S | CBN | Palliative chemotherapy | 2 m | N/S | N | Krishnakumar S, Lakshmi S, Abhyankar D, Biswas J. Loss of antigen-processing  molecules in primary orbital melanoma. Orbit. 2003 Dec;22(4):265-70. |
|  |  |  | F | 35 | N/S | N/S | N/S | CBN | Exenteration&radiotherapy | 12 m | N/S | Y |  |
|  |  |  | M | 43 | N/S | N/S | N/S | CBN | Exenteration&radiotherapy | 18 m | no Metastasis | Y |  |
| 42-43 | Lee^[40]^（2002） | 2 | F | 49 | L | N/S | pink subconjunctival mass lying in conjunctiva | N/S | the mass was explored&orbital radiotherapy | N/S | N/S | N/S | Lee V, Sandy C, Rose GE, Moseley IM, Cree I, Hungerford JL. Primary orbital  melanoma masquerading as vascular anomalies. Eye (Lond). 2002 Jan;16(1):16-20. |
|  |  |  | M | 30 | L | increasing, painless left lower lid mas | N/S | N/S | angiography with superselective catheterisation and embolisation、tumorectomy、orbital radiotherapy、 systemic chemotherapy | 5y | recurrence | Y |  |
| 44 | Leff^[10]^  (1983) | 1 | M | 51 | L | a painlessly mass | N/S | N/S | Excisional biopsy&partial orbital exenteration | 2 y | Metastasis | N | Leff SR, Henkind P. Rhabdomyosarcoma and late malignant melanoma of the orbit. Ophthalmology. 1983 Oct;90(10):1258-60. |
| 45 | Loffler^[5]^（1989） | 1 | M | 27 | R | proptosis | CBN | CBN | exenteration | N/S | N/S | N/S | Löffler KU, Witschel H. Primary malignant melanoma of the orbit arising in a cellular  blue naevus. Br J Ophthalmol. 1989 May;73(5):388-93. |
| 46-47 | Loghavi^[54]^（2014） | 2 | F | 54 | N/S | Proptosis | BN | N/S | N/S | 27.6 m | N/S | Y | Loghavi S, Curry JL, Torres-Cabala CA, Ivan D, Patel KP, Mehrotra M, Bassett R, Prieto VG, Tetzlaff MT. Melanoma arising in association with blue nevus: a clinical and  pathologic study of 24 cases and comprehensive review of the literature. Mod Pathol. 2014 Nov;27(11):1468-78. |
|  |  |  | F | 33 | N/S | Blurry vison | Atypical CBN | N/S | N/S | 85.2 m | metastases | Y |  |
| 48 | M. Schultheiss^[13]^  (2013) | 1 | F | 34 | L | Proptosis& Diplopia | No associated orbital BN or melanosis | N/S | orbital resection&interferon-alpha | 6 m | no Metastasis | Y | Schultheiss M, Rohrbach JM, Süsskind D, Besch D, Spitzer MS. Primäres orbitales  Melanom bei einer 34-jährigen Frau [A thirty-four-year-old woman with primary orbital  melanoma]. Klin Monbl Augenheilkd. 2013 Jul;230(7):680-1. German. |
| 49 | Mahoney^[31]^（2008） | 1 | M | 50 | R | loss of vision&swelling | CBN | N/S | resection via orbital exenteration plus craniotomy with postoperative adjunctive therapy | N/S | N/S | N/S | Mahoney NR, Engleman T, Morgenstern KE. Primary malignant melanoma of the  orbit in an African-American man. Ophthalmic Plast Reconstr Surg. 2008  Nov-Dec;24(6):475-7. |
| 50 | Mandeville^[15]^（2004） | 1 | F | 26 | R | Proptosis | occult nevus of non–blue nevus type | occult nevus of non–blue nevus type | subtotal exenteration of the right orbit& radiotherapy | 2 y | no local recurrence or metastasis | Y | Mandeville JT, Grove AS Jr, Dadras SS, Zembowicz AM. Primary orbital melanoma  associated with an occult episcleral nevus. Arch Ophthalmol. 2004 Feb;122(2):287-90. |
| 51-56 | Mudhar^[46]^（2019） | 6 | M | N/S | L | Reduced VA and pain、proptosis | N/S | N/S | orbital exenteration+Postoperative orbital radiotherapy | N/S | metastases | N/S | Mudhar HS, Doherty RE, Salvi SM, Currie ZI, Tan JH, Sisley K. Genetic Profiling of  Primary Orbital Melanoma: An Analysis of 6 Cases with Clinicopathologic Correlation. Ophthalmology. 2019 Jul;126(7):1045-1052 |
|  |  |  | M | N/S | R | Puffiness around R eye，proptosis | N/S | N/S | orbital exenteration+Postoperative orbital radiotherapy | N/S | no recurrence or metastasis | Y |  |
|  |  |  | F | N/S | L | Left proptosis and left subconjunctival hemorrhage | N/S | N/S | orbital exenteration+Postoperative orbital radiotherapy | 36 m | no recurrence or metastasis | Y |  |
|  |  |  | F | N/S | L | proptosis | N/S | N/S | Systemic palliative support | 8 w | metastases | N |  |
|  |  |  | M | N/S | L | Painless loss of vision、proptosis;restricted eye movements | N/S | N/S | Systemic palliative support | 6 w | metastases | N |  |
|  |  |  | M | N/S | R | Diplopia | N/S | N/S | orbital exenteration | 48 m | No local recurrence and no metastases. Died of unrelated causes 48 months postsurgery | N |  |
| 57 | Odashiro^[11]^（2005） | 1 | F | 43 | R | pain、protrusion&decreased vision | probably arising in a BN | BN | exenteration of the right orbit followed by a course of radiotherapy | N/S | N/S | N/S | Odashiro AN, Arthurs B, Pereira PR, Filho JP, Belfort E, Burnier MN Jr. Primary  orbital melanoma associated with a blue nevus. Ophthalmic Plast Reconstr Surg. 2005  May;21(3):247-8. |
| 58 | Poli^[30]^  （2007） | 1 | M | 60 | R | Proptosis& Diplopia | N/S | N/S | subtotal resection of the tumor | 45 m | no recurrence or metastasis | Y | Poli T, Mora P, Reichegger V, Ricci R, Corradi D, Gandolfi SA, Sesenna E. Chirurgische Behandlung des malignen Orbita-Melanoms: unsere Erfahrung und ein  Bericht über drei Fälle [Surgical management of orbital malignant melanoma: our  experience and a report of three cases]. Klin Monbl Augenheilkd. 2007 Oct;224(10):794-8. German. |
| 59 | Potter^[38]^（2006） | 1 | M | 59 | L | orbital discomfort | no evidence of systemic primary or secondary melanocytic tumor | N/S | Iodine-125 Plaque Radiotherapy&chemotherapy (dacarbazine) | 66 m | Metastasis | Y | De Potter P, Levecq L, Godfraind C, Renard L. Primary orbital melanoma treated  with iodine-125 plaque radiotherapy. Am J Ophthalmol. 2006 Nov;142(5):864-6. d |
| 60 | Radhadevi^[27]^（2013） | 1 | M | 54 | L | Restricted ocular motility, periorbita swelling, loss of ligh perception | nevus of Ota | N/S | N/S | 13 y | recurrence 13 years later; after second treatment no regression at 1 year | Y | Radhadevi CV, Charles KS, Lathika VK. Orbital malignant melanoma associated with  nevus of Ota. Indian J Ophthalmol. 2013 Jun;61(6):306-9. |
| 61 | Ranjit^[37]^（2016） | 1 | F | 87 | R | right eye pain | Oculodermal Melanocytosis&melanoma-associated spongiform scleropathy contiguous | benign melanocytes | orbital exenteration | N/S | N/S | N/S | Ranjit RU, Leyngold IM, Margo CE. Melanoma-Associated Spongiform Scleropathy  in Oculodermal Melanocytosis with Primary Orbital Melanoma. Ocul Oncol Pathol. 2016  Oct;2(4):276-279. |
| 62 | Rice^[43]^  （1990） | 1 | F | 17 | R | Proptosis | CBN with pregnancy | N/S | Not specified | 2 y | no recurrence or metastasis | Y | Rice CD, Brown HH. Primary orbital melanoma associated with orbital  melanocytosis. Arch Ophthalmol. 1990 Aug;108(8):1130-4. |
| 63-73 | Rose^[33]^（2017） | 11 | F | 81 | R | N/S | N/S | N/S | Debulking | 3 m | Metastasis | N | Rose AM, Luo R, Radia UK, Kalirai H, Thornton S, Luthert PJ, Jayasena CN, Verity  DH, Coupland SE, Rose GE. Detection of mutations in SF3B1, EIF1AX and GNAQ in  primary orbital melanoma by candidate gene analysis. BMC Cancer. 2018 Dec  17;18(1):1262. |
|  |  |  | M | 40 | L | N/S | N/S | N/S | Exenteration | 4 m | Metastasis | N |  |
|  |  |  | F | 58 | L | N/S | N/S | N/S | Exenteration | 25 m | Unknown | Y |  |
|  |  |  | M | 45 | L | N/S | N/S | N/S | Debulking + RT（radiotherapy） | 37 m | no Metastasis | Y |  |
|  |  |  | F | 84 | R | N/S | Conjunctival melanosis | N/S | Exenteration + RT | 18 m | Metastasis | N |  |
|  |  |  | M | 45 | L | N/S | Nevus of Ota | N/S | Debulking + RT | 91 m | Metastasis | Y |  |
|  |  |  | F | 47 | L | N/S | Conjunctival nevu | N/S | Debulking + RT | 174 m | Metastasis | N |  |
|  |  |  | M | 46 | R | N/S | N/S | N/S | Debulking +RT | 26 m | no Metastasis | Y |  |
|  |  |  | F | 43 | L | N/S | N/S | N/S | Debulking + RT | 188 m | no Metastasis | Y |  |
|  |  |  | F | 70 | L | N/S | N/S | N/S | Debulking + RT | 22 m | no Metastasis | Y |  |
|  |  |  | M | 55 | R | N/S | N/S | N/S | Debulking + RT | 35 m | Metastasis | Y |  |
| 74  -75 | Rose^[34]^（2017） | 2 | F | 48 | L | N/S | N/S | N/S | Debulking + RT | 19 m | N/S | Y | Rose AM, Luthert PJ, Jayasena CN, Verity DH, Rose GE. Primary Orbital Melanoma:  Presentation, Treatment, and Long-term Outcomes for 13 Patients. Front Oncol. 2017  Dec 18;7:316 |
|  |  |  | F | 60 | R | N/S | N/S | N/S | Debulking + RT | 63 m | N/S | N |  |
| 76 | Shields^[32]^（1993） | 1 | M | 76 | R | blurred vision&epibulbar redness | No evidence of congenital melanocytosis, BN, or CBN | N/S | tumorectomy | 22 m | Metastasis | N | Shields JA, Shields CL, Eagle RC Jr, De Potter P, Oliver GL. Necrotic orbital  melanoma arising de novo. Br J Ophthalmol. 1993 Mar;77(3):187-9. |
| 77 | Shields^[57]^（2003） | 1 | M | 59 | R | N/S | N/S | N/S | N/S | N/S | N/S | N/S | Shields JA, Shields CL. Orbital malignant melanoma: the 2002 Sean B Murphy  lecture. Ophthalmic Plast Reconstr Surg. 2003 Jul;19(4):262-9. |
| 78 | Speakman^[52]^（1973） | 1 | F | 29 | R | Proptosis&diplopia | Naevus of Ota, BN, CBN | N/S | Partial exenteration | 24 m | metastases | N | Speakman JS, Phillips MJ. Cellular and malignant blue nevus complicating  oculodermal melanosis (nevus of Ota syndrome). Can J Ophthalmol. |
| 79-99 | Tellado^[56]^（1996） | 21 | — | mean age at  diagnosis was 42 years (range, 15-84 years) | — | proptosis in L6 (76%), blurred vision in 5 (24%), and dip lopia in 3 (14%) | associated blue nevus in  19 patients (90%), and in 10 patients (47.5%) there was some form of congenital mel anosis | — | In 14 of the 21 patients, orbital exenteration， Seven patients were represented only by excisional biopsies | 4.5 years (range, 1-13 years) | mortality from metastatic tumor occurred in 8 (38%) | 8 died | Tellada M, Specht CS, McLean IW, Grossniklaus HE, Zimmerman LE. Primary orbital  melanomas. Ophthalmology. 1996 Jun;103(6):929-32. |
| 100 | Unal^[25]^  （1992） | 1 | M | 60 | L | Crescent-shaped dark area | nevus of Ota | N/S | N/S | 28 m | no recurrence or metastasis | Y | Unal M, Gunalp I, Deery A, Durak I, Erekul S, Bulay O. Malignant melanoma of the  optic nerve head in a case of oculodermal melanocytosis. Br J Ophthalmol. 1992  May;76(5):313-5. |
| 101 | Wilkes^[44]^（1984） | 1 | M | 18 | N/S | Proptosis&diplopia | Ocular melanocytosis | N/S | Orbital exenteration | N/S | N/S | N/S | Wilkes TD, Uthman EO, Thornton CN, Cole RE. Malignant melanoma of the orbit in a  black patient with ocular melanocytosis. Arch Ophthalmol. 1984 Jun;102(6):904-6. |
